# Misconceptions of Depression in African Americans

**DOI:** 10.3389/fpsyt.2014.00065

**Published:** 2014-06-20

**Authors:** Zohaib Sohail, Rahn Kennedy Bailey, William D. Richie

**Affiliations:** ^1^Department of Psychiatry, Meharry Medical College, Nashville, TN, USA

**Keywords:** depression, under treatment, underserved and misdiagnosed, African Americans, misconception

## Abstract

Major depression is a very common disabling disorder. Although the relationship between race and depression is complex, depression affects all races, all ethnic and geographic locations as well as all age groups. The prevalence of depression in African Americans is controversial, due to the paucity of research. The deficit in the knowledge and skills in treating depression in African Americans have not been adequately addressed so far. Inadequate and insufficient data on African Americans contributes to the problems of under diagnoses, misdiagnosis, and under treatment of depression. This article will highlight the existing problem of depression in Afro American with a focus on diagnostic and treatment issues.

## Introduction

Major depressive disorder (MDD) is the fourth leading cause of disability and a leading cause of non-fatal disease burden (1). It affects one in five persons in the United States and manifests with symptoms that are physiological, emotional, motivational, behavioral, and cognitive as specified by the *Diagnostic and Statistical Manual of Mental Disorders, Fourth Edition* [*DSM-IV*; American Psychiatric Association (APA), 1994].

According to a Survey (2, 3), blacks have lower lifetime rates of MDD and equivalent or lower rates of 12-month MDD compared to non-Hispanic whites.

Blacks have reduced access to mental health services and receive poor quality services as compared to whites (4). National co morbidity survey (NCS) reports blacks despite having lower risk of mood disorder, once diagnosed were more likely to be persistently ill (5).

For defining purpose, African Americans are black people who do not have ancestral ties to the Caribbean. *Caribbean blacks* are also self-identified as black but they have ancestral ties with West Indies or the Caribbean people.

Historically, there is a lack of both diagnostic and treatment studies on depression (6). This lack of studies on depression in African Americans has existed for decades (7). African Americans are underserved, understudied, and misdiagnosed as a group (8). In an Afro American study of psychiatric illness (9), tremendous misdiagnoses were reported.

Sadness, loss of happiness, malaise, loss of motivation, decrease social interaction, disturbed sleeping, eating and sexual habits, difficulty concentrating, synthesizing, and memory losses are few reported symptoms for Depression. Non-traditional symptoms may also be sometimes observed in African Americans such as hypertension (6).

## Discussion

If untreated, depression leads to increased healthcare utilization, which results in emergency room visits from poor quality of life and self-injurious behaviors (10). This in turn causes a negative impact on the economy from increased absenteeism and occupational impairment (11). It disturbs the family by causing failure to thrive symptoms in children of mothers who have co morbid depression and substance abuse disorders.

Currently no specific archetype exists for describing depression etiology. There are several school of thoughts, ranging from organic and physiological factors to biological markers and neurotransmitter deficiencies. The most acceptable theory for depression considers it as a product of complex interaction between psychological and biological factors. There exists a positive relationship between depression and stressful life events and a negative relationship between depression and social support (33). Precipitants, psychological, or somatic, however can be genetic or acquired predispositions.

According to World Health Organization report, “The Global Burden of Disease” (13) depression is considered to be the greatest burden in women when compared to all other diseases. Women in general are reported to have a higher risk of initial episode and earlier onset of depression as compared to men (12). Afro American women have reported three million mental health’s visits each year.

African Americans have a unique history, having been introduced as slaves to this country (8). They have distinctive traditions and practices along with extraordinary individual and collective identities (14). They are aware of their roles as mothers and homemakers but feel guilty when engaging in activities to promote self-development. This role conflict in personal developmental and family survival needs often results in depression in African Americans (15).

Diagnosing and assessing cultural bound symptoms of depression in African Americans is a major concern. Difference in symptom presentations between African Americans and other groups can be determined by cultural based expressions. Cultural competencies are essential for an accurate diagnostic and treatment process designated specifically for each particular racial or ethnic group. A culture-bound syndrome seen in African Americans is a sudden collapse following an episode of dizziness often referred to as “falling-out.” Sleep paralysis, which is characterized by an inability to move while awakening or falling asleep is also sometimes observed in African Americans (16).

According to an estimate nearly half the entire U.S. population will be composed of ethnic and racially diverse people by the year 2052 (6). Within this growing pace of diverse race, it is therefore important to realize that statistics peculiar to Whites is not sufficient to address mental health issues among African Americans (17). The experiences of racism, sexism, and poverty have increased the risk for depression in African Americans.

Prevalence of depression in African Americans is reported to be twice as compared to whites (18).

National study of American life (NSAL) (20) survey reports that the lifetime prevalence of depression is higher for whites (17.9%) than for African Americans (10.4%) and Caribbean blacks (12.9%), but when the course of depression is considered, depression in African Americans persists for longer duration. This measure of persistence was 56.5% for African Americans, 56% for Caribbean blacks, and 38.6% for whites. Thus, major depression is considered as a chronic disorder for blacks. Due to the larger exposure to community and domestic violence, African Americans have higher risk for depression co morbidities, such as substance abuse, generalized anxiety and posttraumatic stress disorders (19).

Despite three decades of research, treatment of depression and its co morbidities still remains a significant public health problem in the United States. The treatment of depression and co morbidities is considered to be the most common and urgent concern faced by mental health professionals these days (21). Definitive studies for depression treatment in African Americans are sparse. The higher risk of persisting depression in blacks calls for a need to focus on treatment modalities and identifying causal factors.

In United States, only 57% of adults with MDD receive treatment (22). It is claimed that successful psychotherapeutic treatments for depression are universal and can be applied equally to all racial and ethnic groups, which may well be an invalid assumption (23).

In a comparison of cognitive psychotherapy and pharmacotherapy treatments for depression, cognitive psychotherapy was found to be equally effective or more effective than pharmacotherapy at initial treatment and follow-up (24). There is a significant reduction in depressive symptoms after 12 sessions of cognitive therapy in African Americans when compared to analytically oriented therapy sessions of similar duration (25).

Holistic therapies address physical and psychological symptoms in both diagnostic and treatment modalities (6). In African Americans, holistic treatments are found to be successful in reducing depressive symptoms (26). An approach to overcome distress and depression is through confronting troubles rather than avoiding them. Try to seek help from family; friends, neighbors, and religious leaders (14). Researchers can take advantage of this particular finding in African Americans to broaden their understanding of the psychological–social strategy.

The influence of religion in the Afro American inventory cannot be ignored. Prayer is a common coping response for African Americans in distress. Today, almost 85% of African Americans describe themselves as “fairly religious” (27) or “very religious” and “religious involvement” can be a moderator to reduce the onset of depression (28).

Ethnicity is a neglected aspect of the heterogeneity of black population (29). Although it is important to study racial differences in treatment outcomes, differences between White/Caucasian Americans and Black/African Americans are not typically studied. When they are reported, the researchers have usually made subsequent comparisons based on samples that are not equally representative.

It is noticed blacks that do have mental healthcare access receive poor quality care as compared to whites (30). Measures should be taken to remove these racial disparities. Providing equal and quality access to all will help eliminate discrimination and disparities. Important gaps in mental health status of African Americans continue to exist even after 150 years of 1840 census (31).

## Conclusion

Blacks, irrespective of ethnicity, are reported considerably more days out of job than the average for persons with Major depression (22). This suggests that when blacks develop depression, it is devastating, persistent in course and has poor prognosis. Clearly, more empirical studies to determine true prevalence rate for depression in African Americans are required to reduce the gap in accessing effective mental healthcare. This will help close the gap in the literature on effective depression treatments for this underserved and understudied group.

This article is not meant to propose guidelines for diagnosis and treatment of depression. It is an attempt to organize our thoughts on misdiagnoses and under treatment of depression in African Americans. Although this lacks empirical data to substantiate its effectiveness, it serves as a means for setting a framework for future thought on this issue. Our goal is to draw attention toward more evidenced-based approaches to broaden understanding of diagnostic and management modalities. Future research also needs to explore how social support systems; psychological funding, acculturation processes, and cultural values could benefit black population improve their mental health (32).

## Conflict of Interest Statement

The authors declare that the research was conducted in the absence of any commercial or financial relationships that could be construed as a potential conflict of interest.

## References

[1] Ustün TB Ayuso-Mateos JL Chatterji S Mathers C Murray CJ Global burden of depressive disorders in the year 2000. Br J Psychiatry (2004) 184:386–92 10.1192/bjp.184.5.386 15123501

[2] Robins LN Regier DA Psychiatric Disorders in America: The Epidemiologic Catchment Area Study. New York, NY: Free Press (1991).

[3] Blazer DG Kessler RC McGonagle KA Swartz MS The prevalence and distribution of major depression in a national community sample: the National Comorbidity Survey. Am J Psychiatry (1994) 151:979–86 8010383 10.1176/ajp.151.7.979

[4] US Department of Health and Human Services. Mental Health: Culture, Race, and Ethnicity: A Supplement to Mental Health: A Report of the Surgeon General Executive Summary. Rockville, MD: US Dept of Health and Human Services, Public Health Service, Office of the Surgeon General (2001).

[5] Breslau J Kendler KS Su M Gaxiola-Aguilar S Kessler RC Lifetime risk and persistence of psychiatric disorders across ethnic groups in the United States. Psychol Med (2005) 35:317–27 10.1017/S0033291704003514 15841868 PMC2748985

[6] Carrington CH Clinical depression in African American women: diagnoses, treatment, and research. J Clin Psychol (2006) 62(7):779–91 10.1002/jclp.20289 16703605

[7] Das AK Olfson M McCurtis HL Weissman MM Depression in African Americans: breaking barriers to detection and treatment. J Fam Pract (2006) 55(1):30–9 16388764

[8] Baker FM Bell CC Issues in the psychiatric treatment of African Americans. Psychiatr Serv (1999) 50(3):362–8 10096640 10.1176/ps.50.3.362

[9] Adebimpe VR Overview: white norms and psychiatric assessment of black patients. Am J Psychiatry (1981) 138:279–85 7008631 10.1176/ajp.138.3.279

[10] Greenberg GA Rosenbeck RA Changes in mental health service delivery among blacks, whites and Hispanics in the Department of Veterans Affairs. Adm Policy Ment Health (2003) 31:31–43 10.1023/A:1026096123010 14650647

[11] Wells K Sherbourne C Schoenbaum M Ellner S Suan N Miranda N Five year impact of quality improvement for depression: results of a group-level randomized controlled trial. Arch Gen Psychiatry (2004) 61:378–86 10.1001/archpsyc.61.4.378 15066896

[12] McGrath E Keita G Strickland B Russo N, editors. National Depression Summit. Washington, DC: American Psychological Association (2001).

[13] Murray C Lopez A Global Burden of Disease: A Comprehensive Assessment of Mortality and Disability from Diseases, Injuries, Risk Factors in 1990 and Projected to 2020. Cambridge, MA: Harvard University Press (1996).

[14] Browman C Coping with personal problems. In: Neighbors HV Jackson JS, editors. Mental Health in Black Americans. Thousand Oaks, CA: Sage (1997). p. 117–29

[15] Warren BJ Depression in Afro American women. J Psychosoc Nurs Ment Health Serv (1994) 32(3):29–33 8196018 10.3928/0279-3695-19940301-09

[16] Bell C Dixie-Bell A Thompson B Further studies on the prevalence of isolated sleep paralysis in black subjects. J Natl Med Assoc (1986) 78:649–659 3746934 PMC2571385

[17] US Department of Health and Human Services. United States Census Report. Rockville, MD: US Department of Health and Human Services (1999).

[18] Kessler RC The national co morbidity survey: preliminary results and future directions. Int J Methods Psychiatr Res (1995) 5:139–51

[19] Lawson WB Carrington CH Media interview: depression and PTSD in African Americans in the DC community: National Depression Screening Day at Howard University Hospital. Psychiatr News (2003) 38:14

[20] Jackson JS Torres M Caldwell CH Neighbors HW Nesse RM Taylor RJ The national survey of American life: a study of racial, ethnic, and cultural influences on mental disorders and mental health. Int J Methods Psychiatr Res (2004) 13:196–207 10.1002/mpr.177 15719528 PMC6878295

[21] McGrath E Keita G Strickland B Russo N National Depression Summit. Washington, DC: American Psychological Association (2001).

[22] Kessler RC Berglund P Demler O Jin R Koretz D Merikangas KR National comorbidity survey replication. The epidemiology of major depressive disorder. J Am Med Assoc (2003) 289:3095–105 10.1001/jama.289.23.3095 12813115

[23] Lisa Cooper-Patrick MPH Powe NR Jenckes MW Gonzales JJ Levine DM Ford DE Identification of patient attitudes and preferences regarding treatment of depression. J Gen Intern Med (1997) 12(7):431–8 10.1046/j.1525-1497.1997.00075.x 9229282 PMC1497133

[24] Hollon SD DeRubeis RJ Shelton RC Amsterdam JD Salomon RM O’Reardon JP Prevention of relapse following cognitive therapy vs. mediations in moderate to severe depression. Arch Gen Psychiatry (2005) 62:417–422 10.1001/archpsyc.62.4.417 15809409

[25] Carrington CH Treating depression in black women. Urban Res Rev (1981) 7(3):7–11

[26] Taylor J Henderson D Jackson BB A holistic model for understanding and predicting depressive symptoms in African American women. J Community Psychol (1991) 19(4):306–20 10.1002/1520-6629(199110)19:4<306::AID-JCOP2290190403>3.0.CO;2-Z

[27] Taylor RE Chatters LM Taylor RJ Levin JS Lincoln KD Mental health services in faith communities: the role of clergy in black churches. Soc Work (2000) 54:73–87 10.1093/sw/45.1.73 10634088

[28] Watlington CG Murphy CM The roles of religion and spirituality among Afro American survivors of domestic violence. J Clin Psychol (2006) 62:837–57 10.1002/jclp.20268 16703603

[29] Williams DR Jackson JS Race/ethnicity and the 2000 census: recommendations for Afro American and other black populations in the United States. Am J Public Health (2000) 90:1728–30 10.2105/AJPH.90.11.1728 11076240 PMC1446397

[30] Smedley BD Stith AY Nelson AR, editors. Unequal Treatment: Confronting Racial and Ethnic Disparities in Health Care. Washington, DC: National Academies Press (2003). 25032386

[31] Ellis G Mental Illness in the Afro American Community (2011). Available from: http://www.blackpressusa.com/news/Article.asp?SID=4&TitleDepartment&NewID=8812 06/17/11

[32] Williams DR Neighbors HW Social perspectives on mood disorders. In: Kupfer DJ Schatzberg AF Stein DJ, editors. Textbook of Mood Disorders. Arlington, VA: American Psychiatric Publishing Inc (2006). p. 145–58

[33] Warren BJ Depression, stressful life events, social support, and self-esteem in middle class African American women. Arch Psychiatr Nurs (1997) 11(3):107–17 10.1016/S0883-9417(97)80033-7 9193115

